# A model for foreign exchange markets based on glassy Brownian systems

**DOI:** 10.1371/journal.pone.0188814

**Published:** 2017-12-05

**Authors:** M. A. Sánchez-Granero, J. E. Trinidad-Segovia, J. Clara-Rahola, A. M. Puertas, F. J. De las Nieves

**Affiliations:** 1 Department of Mathematics, University of Almería, Almería, Spain; 2 Department of Economics and Business, University of Almería, Almería, Spain; 3 Department of Applied Physics, University of Almería, Almería, Spain; 4 i2TiC Multidisciplinary Research Group, Open University of Catalonia, Barcelona, Spain; East China University of Science and Technology, CHINA

## Abstract

In this work we extend a well-known model from arrested physical systems, and employ it in order to efficiently depict different currency pairs of foreign exchange market price fluctuation distributions. We consider the exchange rate price in the time range between 2010 and 2016 at yearly time intervals and resolved at one minute frequency. We then fit the experimental datasets with this model, and find significant qualitative symmetry between price fluctuation distributions from the currency market, and the ones belonging to colloidal particles position in arrested states. The main contribution of this paper is a well-known physical model that does not necessarily assume the independent and identically distributed (i.i.d.) restrictive condition.

## Introduction

Since Fama [1] showed that the normal distribution does not fit the empirical distribution of stock market returns, which is leptokurtic and has heavy tails, financial market distributions have become a topic in financial literature. According to McDonald [2], the normal and the log-normal distributions were widely used mainly for two reasons: the estimation of their parameters becomes relatively simple and provides appropriate descriptive models in most cases. Today it is not easy to summarize research papers proposing different distributions in financial markets around the world. A distribution widely used in the literature has been the Student one. This distribution seems to be helpful for two reasons: first, it is adequate in resolving distribution tails and second, when the number of degrees of freedom is greater than 30, the Student distribution converges to a normal one.

Alternatively, Mandelbrot [3] proposed to replace the Brownian motion approach resulting from the normal distribution, by a model based on a symmetric stable Levy motion of parameter *α* < 2. After Mandelbrot’s paper, the Levy distribution family became very popular as they account for asymmetry and heavy tails. For example, Press [4] introduced an exponential Levy process model with a non-stable distribution based in a superposition of Brownian motion and an independent compound Poisson process with normally distributed jumps. Madan and Seneta [5] proposed a Levy process with gamma variance distributed increments and Barndorff [6] used the family of generalized hyperbolic distributions. Later, Eberlein and Keller [7] introduced an exponential hyperbolic Levy motion, Koponen [8] employed the geometric stable laws, Kozubowski and Panorska [9] considered the multivariate geometric stable distribution or Kozubowski and Podgorski [10] proposed the asymmetric Laplace one, which is a subclass of geometric stable distributions. Note that such distributions allow for asymmetry, they have finite moments of any order, their densities have explicit forms and the estimation of their parameters is easy.

As final remark about the use of the stable family in finance, it must be mentioned that Kim et al. [11] developed the modified tempered stable and Koponen [12], Boyarchenko and Levendorskii [13], Carr et al. [14] introduced the classical tempered stable distribution.

The use of stable families in finance has been conditioned mainly due to the difficulty to estimate the parameters which are well-known only in limiting cases. Another problem associated with this family of distributions is the overestimation of tail indices when samples are not large enough, the infinite second moment and that they do not account for the peakedness around the origin often seen in stock returns.

A different contribution was presented by Login [15] who proposed a Frechet distribution to model extreme returns. Clark [16] and Epps and Epps [17] introduced in foreign exchange markets the so-called mixture distribution hypothesis (MDH) by assuming the strong correlation between trading volume and volatility of exchange rates. In this line, Tauchen and Pitts [18] derived the joint distribution of daily price changes and transactions volumes from a model of intraday equilibrium price changes and intraday volumes. Recent contributions are due to Masoliver et al [19], where a stochastic model for high frequency data in the Standard and Poor’s 500 cash index is presented, Masoliver and Montero [20] where the authors introduce a continuous time random walk model to model the US dollar/Deutsche Mark future and finally Masoliver and Perello [21] where an Exponential Ornstein-Uhlenbeck stochastic volatility model is proposed, which is able to capture multi-scale behavior in the volatility autocorrelation.

Most of the models developed so far propose different distributions considering stylized facts in financial data. The advance in computation methods have allowed researchers to use more complex distributions with more flexible parameters, thus better descriptions of empirical data have been achieved. However, a major problem still remains: estimations are not stable enough in time and the independent and identically distributed (iid) hypothesis persists. In fact, a single functional form is often not able to depict the whole distribution spectrum [22, 23]. In view of such scenario, it is often the case that a pieced functional form is considered in order to quantitatively model financial distributions, where usually a Gaussian distribution is taken when focusing on the central peak of the distribution, while Levy flights are the ones employed in describing heavy distribution tails [24].

In our approach, we present a model which is characteristic to the dynamics of many different physical particle systems, such as atomic glasses, undercooled fluids, granular matter, polymer and colloidal gels, … [25]. All of these systems have in common that their global dynamics is very slow, or even arrested; density fluctuations take very long time to relax, showing viscoelastic behaviour. Microscopically, this is rationalized considering that particles are *caged* by their own neighbors.

Recall that in fluids at high temperature or gasses, fluctuations in the density can relax very fast because molecules are highly movable, whereas in solids, the motion of single molecules is strongly hindered, disabling the relaxation of local stresses. In undercooled fluids, an intermediate situation is found. At short times, the rattling of the particles inside the cage results in short time dynamics, which saturates when the cage is explored, while long time diffusion requires cooperativity of the neighbors to allow the escape of the particle. This is also interpreted physically by using a free energy hyper-surface, which, in supercooled fluids or glasses, has multiple shallow minima: the vibrations within a single minimum correspond to the rattling in the cage, and long time dynamics is described as jumps from one minimum to another one.

Different models have been developed to describe the dynamics of these systems, and in particular hopping models have been reported. However, please observe that the existing literature concerns models where important restrictions, such as restricted number of investors, restricted market volume or restricted positions, must be considered [26]. Also, other models do not provide a fundamental scope [27], such as the one proposed in this work. Here price fluctuations from the currency exchange market are depicted through a physical model proven valid for a wide variety of physical systems, for example atomic and molecular ones. Namely, we have focused on a particular model proposed by Chaudri et al. [25], where two time scales are considered for the jump from minimum to minimum, and it has been extended to the study of currency exchange rates. We have found that such model is an excellent description to financial distributions, such is the case to the Euro—US dolar [28], among other currencies presented in this work. Noteworthly, this analysis does not assume the data to be independent and identically distributed, i.i.d. Furthermore, the parameters that are employed in the model keep physical significance and therefore, not only a single functional form describing the full distribution range has been found, but even more, the physical understanding that underlies the model allows us to rationalize financial markets.

Here it is important to remark that our approach is as well useful from an applied point of view as it allows developing analysis and instruments aimed at market operations. Furthermore, it must be pointed out that the already mentioned combination of Gaussian and Levy distributions are often used by hedge funds and investors in general in order to monitor market activity and develop investing strategies. Within this regard, the model presented in this manuscript can be very effective because a single description is proposed, where for example, the probability of price changes and its range can be statistically determined. However, we would like to emphasize that our main contribution is the extrapolation of a well known model used for supercooled or arrested states in glassy physics to study the behaviour of foreign exchange rate markets.

This paper is structured as follows: section 2 introduces some of the most important findings of financial literature of foreign exchange rate markets; section 3 describes our physical model; section 4 shows the results of the fits in different currencies and finally section 5 contains the main conclusions.

## Foreign exchange markets: A market characterization

In this section we summarize from Sarno and Taylor [29] some characteristics of the microstructure of the foreign exchange market which are relevant to our model.

The foreign exchange market presents some special characteristics over other financial markets. It is a decentralized market in which not all dealer quotes are observable, since trades need not be disclosed and transaction does not occur with just one institution, so different prices can be transacted at the same time. This implies that order flow is not a reliable source of data. Additionally, market makers are responsible for most of the trading volume and this role is assumed mainly by commercial and investment banks. On the other hand, foreign exchange markets are the clearest example of continuous market because it is open 24 hours a day except weekends, and trading volume is the most extensive around the world. This feature explains why the foreign exchange market is among the most efficient ones.

The role assumed by investment banks is for several authors [30–32] the reason why market evolution is largely unexplained by movements from macroeconomic fundamentals. Many works in the field also do not assume that only public information is relevant to exchange rates [33].

Financial literature also shows (see [33–35]) that time aggregated order flow variables could be more powerful than macroeconomic variables in explaining the exchange rate behavior.

A standard assumption in foreign exchange markets has been that expectations are rational, but the literature provides evidence of risk premia and rejects the rational expectation hypothesis. It seems clear by most of the authors that the formation process used by agents in the foreign exchange market is likely to be more complex than other markets, and that heterogeneity of expectations is crucial [36]. We would like to remark the work of Frankel and Froot [37] which presents a formal model of agent expectations in the foreign exchange market, where agents are classified as chartists, fundamentalists and portfolio managers. They conclude that the value of a currency can then be driven by the decisions of portfolio managers who consider a weighted average of the expectations of fundamentalists and chartists. Here we find another crucial point in exchange rate literature, namely, the role of analysts.

The discrepancy between short and long run exchange rate expectations could be attributable to market participants that use chartist analysis for short run whereas the technique used for long run is fundamental analysis or conventional portfolio models. Evidences are given by Allen and Taylor [38], Taylor [36], Menkhoff [39, 40] and Cheung and Wong [41, 42], Cheung and Chinn [43]. All authors conclude that economic fundamentals will win in the long term and that short term price movements may be dominated by chartist analysis.

## Introducing the model

In Clara et al. [28], a model borrowed from physical glasses, that has proven successful when describing data from experiments and simulations [25], is introduced to describe the fluctuations of the euro—US dollar (EURUSD) currency pair. Here, we test such model with many different currency pairs: Pound sterling—Japanese yen (GBPJPY), Australian dollar—Canadian dollar (AUDCAD), New Zealand dollar—Singapur dollar (NZDSGD), US dollar—Mexican peso (USDMXN), Euro—Swiss franc (EURCHF), Pound sterling—Polish zloty (GBPPLN), US dollar—Chinese yuan (USDCNH), US dollar—Hong Kong dollar (USDHKD) and US dollar—Turkish lira (USDTRY). We aim to resolve if the model can be applied to the currency market in general and not only to the EURUSD case. Therefore, we have selected different currency pairs in order to test such approach. We use data with a frequency of 1 minute for periods of one year, from 2010 to 2016 (depending on data availability).

We focus on price fluctuations in the currency pair, and study the distribution of the logarithmic return (in short, log-return) for a given lag time *τ*, *r*(*τ*) = log(*p*(*t*_0_ + *τ*)/*p*(*t*_0_)). (Alternatively, the log-return can be also defined as *r*(*τ*) = log(*p*(*t*_0_)/*p*(*t*_0_ − *τ*))). Probability distribution functions (pdfs) from currency rates exhibit a symmetric profile with long tails, specially for small values of *τ*. These pdfs are common to all the currencies under study and the overall profile is about the same. Note that in [28] the bare price fluctuation is used, but in this work we study the log-return instead, thus, we can compare among different pairs.

Typically, the study of financial log-return distribution (see the introduction) is modelled by using a distribution that provides a good description of experimental data, but without any other significant meaning. Other authors make strong assumptions about the number and the kind of agents. The model we use is based on a model introduced to study particle displacements in physical glasses, where every particle is ideally caged by its own neighbors, restricting the structural relaxation of the whole system. Thermal fluctuations, however, allow particles to jump from one cage to another, on a large time scale.

**Fig 1:**
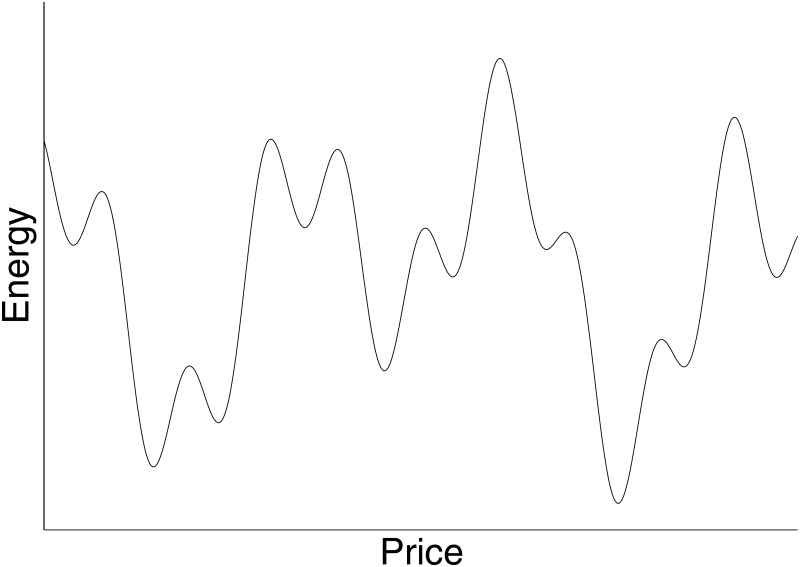
Schematic representation of the energy landscape. Schematic representation of the energy landscape as a function of the price.

The model proposed here is based on the description of the free energy landscape of supercooled liquids as a hypersurface composed by many shallow minima, where the system is transiently trapped before a jump is attempted to a different minimum (in contrast, in fluids, the landscape is almost flat, whereas in crystalline solids, it has a deep absolute minimum, corresponding the crystal structure) [44]. The extrapolation to financial markets proposed here assumes that a given currency pair moves in a free energy with many shallow minima, as shown schematically in Fig 1. Two different processes can be immediately identified: i) vibrations within a single minimum, and ii) jumps to other minima. Even more, because the system is expected to be trapped longer in deeper minima, it can be assumed that the first jump out of this deep minimum has a longer waiting time, whereas subsequent jumps will occur faster, as the system is exploring other minima. Our model takes into account all of these processes.

The vibrations inside a local minimum is described by an Ornstein-Uhlenbeck process, given by

$$\begin{array}{c} f_{vib}\left(r,t\right)=\sqrt{\alpha /2\pi D(1-e^{-2\alpha t)})}\exp\left\{-\alpha r^{2}/2D\left(1-e^{-2\alpha t}\right)\right\} \end{array}$$ (1)

with *r* the logarithm of the return, as defined previously, *D* the diffusion coefficient and *α* = *D*/*l*^2^, with *l* the size of the cage [45–47]. This process depicted originally a particle describing Brownian motion with a linear central force pulling it towards its origin, and has been adapted to a one-dimensional motion.

Long range jumps are possible on a larger time scale, according to a Gaussian distribution:

$$\begin{array}{c} f_{jump}\left(r\right)={\left(2\pi d^{2}\right)}^{-3/2}exp\left(-r^{2}/2d^{2}\right) \end{array}$$ (2)

where *d* is the typical size of the jumps. As mentioned previously, different waiting times are considered for the first and all other subsequent jumps. Both probabilities are drawn from an exponential distribution. For the first jump, $\phi_{1}\left(t\right)=\tau_{1}^{-1}exp\left(-t/\tau_{1}\right)$ has a typical time *τ*_1_, while subsequent jumps occur faster according to $\phi_{2}\left(t\right)=\tau_{2}^{-1}exp\left(-t/\tau_{2}\right)$, with a time scale *τ*_2_ < *τ*_1_.

The overall log-return distribution, *G*(*r*, *t*) depicts the probability of a log-return *r*, at time span *t*, and it is calculated in the Fourier-Laplace domain, *G*(*q*, *s*). *G*(*r*, *t*) is recovered by back transforming to the log-return–time domain as:

$$\begin{array}{c} G\left(r,t\right)=\tau_{1}f_{vib}\left(r\right)\phi_{1}\left(t\right)+FT^{-1}[{\tilde{f}}_{vib}\left(q\right)\tilde{f}\left(q\right)\tau_{2}\times \\ \times \frac{\exp\left\{\left(\tilde{f}\left(q\right)-1\right)t/\tau_{2}\right\}-\exp\left(-t/\tau_{1}\right)}{\tau_{2}-\tau_{1}+\tilde{f}\left(q\right)\tau_{1}}] \end{array}$$ (3)

Here $\tilde{f}\left(q\right)={\tilde{f}}_{vib}\left(q\right){\tilde{f}}_{jump}\left(q\right)$, $\tilde{f}\left(q\right)$ is the Fourier transform of function *f*(*r*), *q* is the conjugate variable of log-return *r* in the Fourier space and *FT*^−1^ denotes the Inverse Fourier Transform.

In physical glasses, this model allows the identification of systems with fast or slow dynamics—high or low temperature fluid, respectively [25]. In a high temperature fluid, the relaxation of local fluctuations is fast because the molecules or particles are highly mobile, whereas in a supercooled fluid this relaxation is much slower. In the model, the former is identified by *τ*_1_ ≈ *τ*_2_ and *l* ≈ *d*, whereas for low temperature fluids, *τ*_1_ ≫ *τ*_2_ and *d* ≫ *l*. Within the picture of the energy landscape, the former indicates that there are no independent basins, and movement of the system through this hypersurface is rather smooth and continuous. On the other hand, *τ*_1_ ≫ *τ*_2_ and *d* ≫ *l* signal the presence of independent minima, with a highly restricted motion. For very long lag times (*τ* ≫ *τ*_1_), the theoretical pdf indeed crosses over to a Gaussian distribution, because the price has experienced many jumps with time scale *τ*_2_, and the contribution from the initial jump can be neglected. This is indeed observed in the experimental pdf [28].

To estimate the parameters of the model, we use the absolute moments as follows: we try to optimize an objective function *f*(*params*) = ∑_*o*∈*O*_ *f*_*m*_(*o*), where *O* is a set with the selected order of the moment, in our case *O* = {0.1,1,2,3,4} and *f*_*m*_(*o*) is the difference in the absolute moment of order *o* between the empirical data and the theoretical distribution corresponding to the given parameters and for a wide range of lag times: *f*_*m*_(*o*) = ∑_*t*∈*T*_|*log*(*mom*_*e*_(*t*))−*log*(*mom*_*t*_(*t*))|/*o*, where *mom*_*e*_(*t*) = < |*r*_*i*_(*t*)|^*o*^>, *mom*_*t*_(*t*) = *E*(|*r*(*t*)|^*o*^), with *r*_*i*_(*t*) the empirical log-return with lag *t* and *r*(*t*) the theoretical log-return with lag *t* for the given parameters, and *T* is a selection of time lags, in our case {[*e*^*z*^]: *z* = 0, 0.5, 1.0, 1.5…,7}.

Since moments of low (high) order give weight to the maximum (tails) of the distribution, our selection of moments for the fitting is aimed to obtain a good fitting in all regions of the pdf, including the mean (*o* = 1) and variance (*o* = 2). Note that we use the absolute moment of order 0.1 to improve the fit around the mode, since the empirical distribution is quite peaked. On the other hand, for some pairs we found few extreme values of the distribution that we do not consider when fitting the parameters, since these values affect too much the absolute moment while are not so representative of the overall distribution. Additionally, by fitting these moments, we ensure to capture the most relevant features of the experimental pdf, such as its skewness (given by the third moment), or the kurtosis (fourth moment).

The final goodness of the fitting is tested by the maximum difference between the experimental and theoretical cumulative distribution function (CDF), calculated as

$$\begin{array}{c} Er=\text{sup}\{|g\left(x\right)-g_{exp}\left(x\right)|:x\in ]-\infty ,\infty [\} \end{array}$$ (4)

where $g\left(x\right)=\int_{-\infty }^{x}drG\left(r\right)$ and $g_{exp}\left(x\right)=\int_{-\infty }^{x}drG_{exp}\left(r\right)$, with *r* the log-return, are the CDF. Because the pdf is normalized, *g*(*x*) grows monotonically from 0 to 1. In most cases, the maximum difference is below 0.05, showing the good quality of the fitting; particular cases are discussed below.

**Fig 2:**
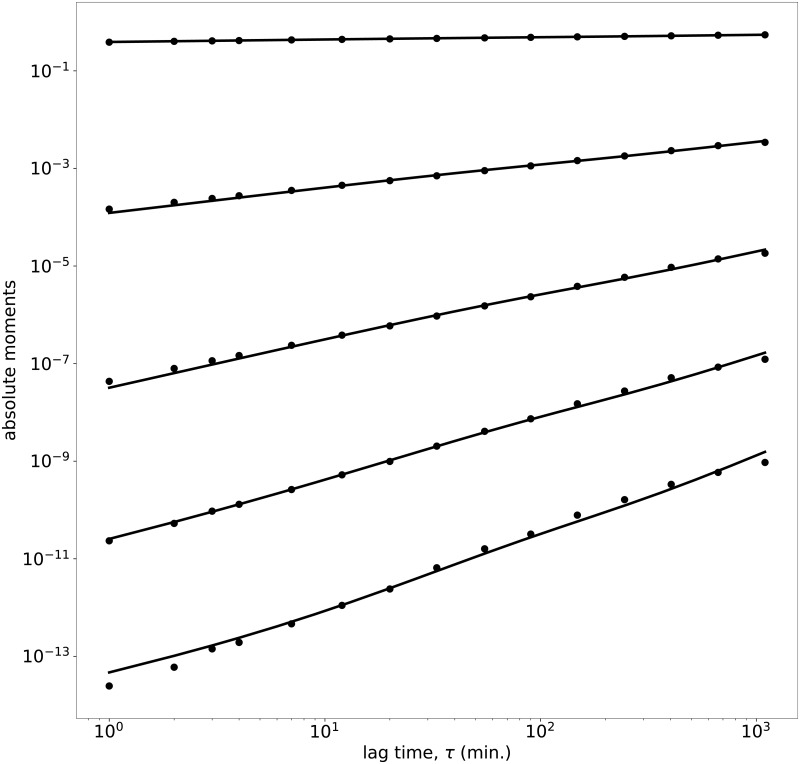
Absolute moments. Absolute moments of order 0.1, 1, 2, 3, 4 (from top to bottom) of AUDCAD for the year 2010.

**Fig 3:**
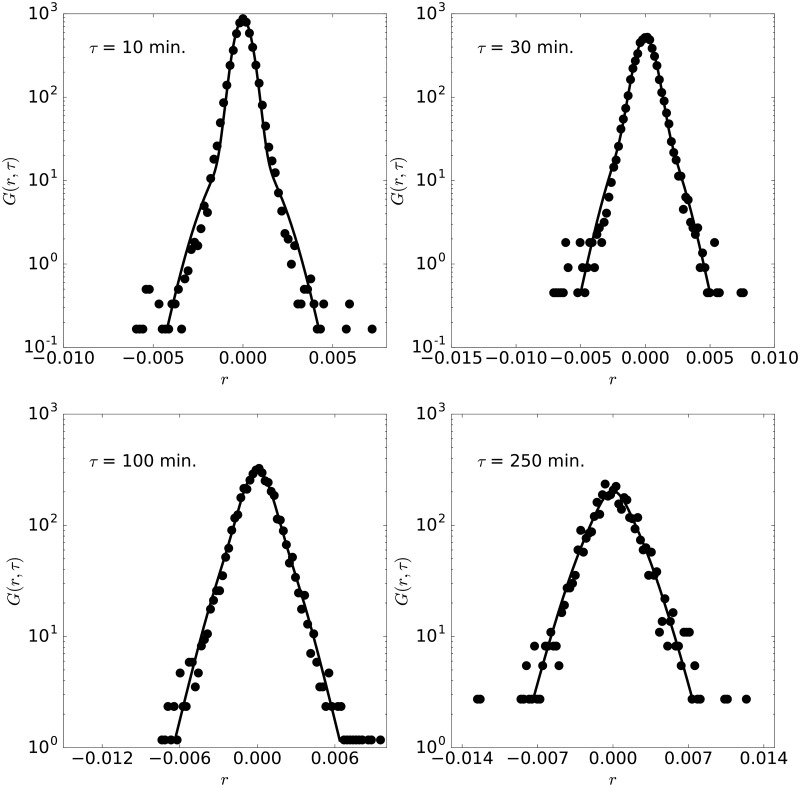
Pdf of AUDCAD. Pdf of AUDCAD with lag times 10, 30, 100 and 250 minutes, for the year 2010. The lines are the pdfs obtained from the moment fittings shown in Fig 2.

A typical fitting is analyzed in Fig 2 by presenting the absolute moments of order 0.1, 1, 2, 3, 4 of the AUDCAD currency pair for the year 2010 (taken as an example), as well as the absolute moment of the fitted model. The fitting is very good for all moments, in particular for the moment of order 0.1. This guarantees that the distribution calculated from the model reproduces the experimental one, as shown below (see Fig 3).

## Results

The fitted probability density functions for lag times of 10, 30, 100 and 250 minutes are shown for the AUDCAD currency pair in Fig 3. As expected from the comparison of the moments, the experimental distributions can be fitted by our model with good quality. In particular, the different trends of the pdf for short and large log-returns are correctly captured for all lag times. The maximum distance between the experimental and theoretical CDF is in this case below 2% in all cases, confirming the validity of the model to reproduce the experimental pdf. Recall that the same set of parameters {*D*, *l*, *d*, *τ*_1_, and*τ*_2_} is used for all the pdfs shown in the figure, demonstrating the capability of the model presented here.

The fitting parameters are given in Table 1 (top row), with the parameters for all other fittings discussed below. Note that *τ*_1_ and *τ*_2_ are very similar, which, within the model, indicates that the dynamics is very fast, similar to a fluid at high temperature (in any case, recall that *τ*_1_ ≥ *τ*_2_, as imposed by the model). The parameters *l* and *d* indicate the size of the cage and length of jumps, as previously explained. For 2010, the model interprets the AUDCAD exchange rate as vibrating in the range ca. 0.14% and jumps out of this “cage”, within a time scale of two hours approximately, to a value 0.115% apart. The similarity of *l* and *d* confirms that the dynamics of this system is fast, i.e. there are no independent minima in the energy landscape (in systems with slower dynamics, it is expected that the separation between the minima is larger than the average size of the basins, *l* ≪ *d*).

**Table 1:** Fitting parameters of the model for different currency pairs and years.

Pair	year	*l*	*d*	*τ*_1_ (min.)	*τ*_2_ (min.)	*D* (min.^−1^)
AUDCAD	2010	0.001378	0.001150	123.66	119.67	1.070e-08
AUDCAD	2011	0.001025	0.000911	73.60	72.42	0.928e-08
AUDCAD	2012	0.000515	0.000555	58.00	50.61	0.375e-08
AUDCAD	2013	0.001197	0.001008	167.74	112.56	0.683e-08
AUDCAD	2014	0.001244	0.001173	197.17	166.64	0.533e-08
AUDCAD	2015	0.001432	0.001379	159.00	137.61	0.857e-08
AUDCAD	2016	0.001451	0.001168	187.02	167.97	0.805e-08
GBPJPY	2010	0.002530	0.002147	228.19	182.45	2.200e-08
GBPJPY	2011	0.004408	0.002613	1011.87	1011.87	1.390e-08
GBPJPY	2012	0.001171	0.000891	106.88	75.45	0.866e-08
GBPJPY	2013	0.001578	0.001279	122.47	117.17	1.150e-08
GBPJPY	2014	0.001022	0.001067	190.11	112.26	0.541e-08
GBPJPY	2015	0.001232	0.001119	145.45	99.30	0.793e-08
GBPJPY	2016	0.034523	0.002854	174.55	174.55	0.807e-08
EURUSD	2010	0.001131	0.001019	79.86	51.84	1.13e-08
EURUSD	2011	0.002063	0.001382	205.50	144.19	1.24e-08
EURUSD	2012	0.000772	0.000865	96.15	60.43	0.55e-08
EURUSD	2013	0.000705	0.001126	163.32	112.11	0.40e-08
EURUSD	2014	0.000271	0.000738	71.81	71.81	0.10e-08
EURUSD	2015	0.000853	0.001394	93.25	85.68	0.80e-08
EURUSD	2016	0.001119	0.001506	243.88	243.88	0.52e-08
NZDSGD	2010	0.001295	0.001532	109.99	109.99	2.110e-08
NZDSGD	2011	0.001172	0.001769	154.61	154.61	2.370e-08
NZDSGD	2012	0.000623	0.000587	47.91	44.46	0.692e-08
NZDSGD	2013	0.000757	0.000867	55.04	46.91	0.927e-08
NZDSGD	2014	0.000500	0.000753	49.58	49.58	0.737e-08
NZDSGD	2015	0.002569	0.002075	396.99	396.70	1.240e-08
NZDSGD	2016	0.000934	0.001166	84.01	84.01	1.060e-08

**Fig 4:**
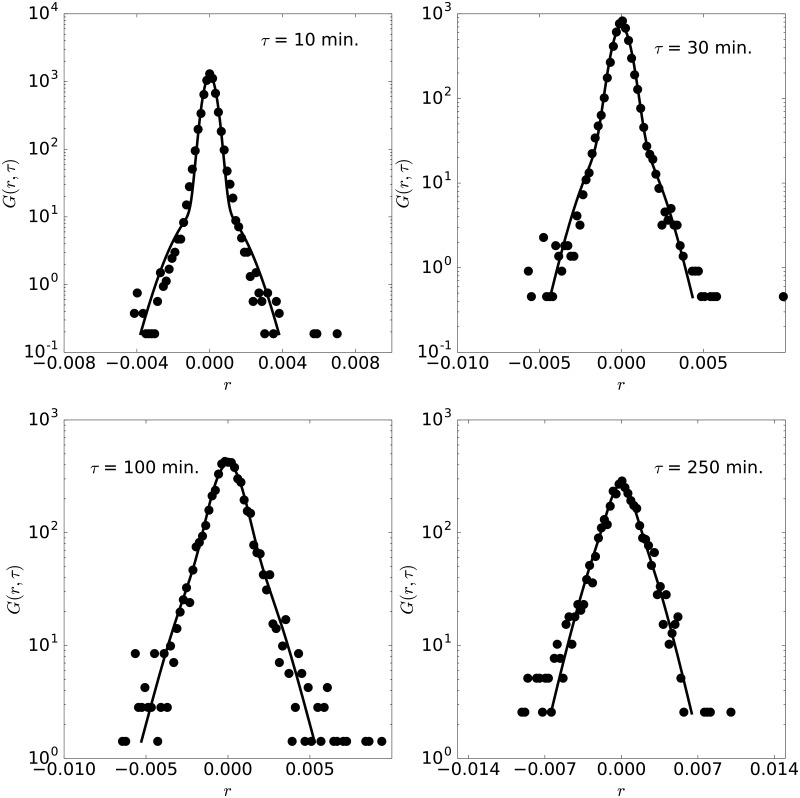
Pdf of AUDCAD. Pdf of AUDCAD with lag times 10, 30, 100 and 250 minutes, for the year 2014.

Fittings of similar accuracy are obtained for all other years of this pair, as illustrated in Fig 4, corresponding to the pdf of the year 2014. The parameters for all years considered here are indicated in the Table 1 (first block). Note that the time scales from all years, *τ*_1_ ≳ *τ*_2_, which, as discussed previously, indicates that AUDCAD currency pair displays fast dynamics, similar to a fluid at high temperature. The time scale, however, varies from the initial magnitude of two hours within a range of one hour in these years. The length scales, for the cage and jumps, evolve also in this period but stay within the range of [0.05%,0.15%]. For all these years and lag times, the maximum separation between the experimental and theoretical CDF is below *Er* = 0.04. Note that to the AUDCAD currency pair and as well to all other currencies presented in this work (see Tables 1 and 2), *τ*_1_ and *τ*_2_ are of about one to three hours. Such hallmark can be qualitatively understood. Short term investors and traders operate in a time range of few hours, and thus determine the short-time dynamics of currency markets. Also, they act synchronized with other financial markets and floors (as operations are not restricted to one market), in particular the NYSE, even by following their schedules related to low (night) and high (morning) activity. Therefore, these agents place operations according to a daily schedule, at particular moments, which impacts market dynamics and the location of stronger or weaker price fluctuations in time, thus setting the magnitude of *τ*_1_ and *τ*_2_.

**Table 2:** Fitting parameters of the model for different currency pairs and years.

Pair	year	*l*	*d*	*τ*_1_ (min.)	*τ*_2_ (min.)	*D* (min.^−1^)
USDMXN	2010	0.001443	0.001761	168.82	168.82	1.50e-08
USDMXN	2011	0.001040	0.001116	157.03	157.01	0.94e-08
USDMXN	2012	0.000875	0.001042	71.28	56.64	0.93e-08
USDMXN	2013	0.001193	0.001383	135.10	95.71	0.81e-08
USDMXN	2014	0.000414	0.000959	82.88	82.78	0.41e-08
USDMXN	2015	0.000590	0.001122	54.74	53.71	0.66e-08
USDMXN	2016	0.001422	0.002411	153.06	153.06	1.66e-08
EURCHF	2011	0.000812	0.002228	168.33	167.51	0.876e-08
EURCHF	2012	0.000060	0.000393	165.16	165.16	1.660e-08
EURCHF	2013	0.000453	0.000749	159.42	159.41	0.193e-08
EURCHF	2014	0.000227	0.000373	213.54	213.54	0.044e-08
EURCHF	2015	0.000546	0.001123	97.98	97.98	0.739e-08
EURCHF	2016	0.000441	0.000829	159.70	159.69	0.269e-08
GBPPLN	2015	0.001070	0.001243	94.18	93.47	0.99E-08
GBPPLN	2016	0.000948	0.001681	108.66	108.66	1.25E-08
USDCNH	2015	0.000480	0.000603	174.72	174.70	0.132E-08
USDCNH	2016	0.000261	0.000474	108.46	108.46	0.090E-08
USDHKD	2010	0.000019	0.000095	79.49	79.49	0.213E-08
USDHKD	2011	0.000020	0.000103	73.30	73.30	0.395E-08
USDHKD	2012	0.000017	0.000066	173.99	173.99	0.153E-08
USDHKD	2013	0.000024	0.000046	168.15	168.14	0.163E-08
USDHKD	2014	0.000021	0.000091	362.77	362.77	0.004E-08
USDHKD	2015	0.000020	0.000102	408.48	405.39	0.002E-11
USDHKD	2016	0.000039	0.000189	165.46	165.41	0.011E-10
USDTRY	2011	0.000525	0.001325	51.71	51.40	2.710E-08
USDTRY	2012	0.000389	0.000930	72.93	72.93	0.605E-09
USDTRY	2013	0.000396	0.001174	76.95	76.95	0.422E-08
USDTRY	2014	0.000904	0.001993	179.11	178.93	0.424E-08
USDTRY	2015	0.001185	0.001747	121.99	103.07	1.030E-08
USDTRY	2016	0.000851	0.001733	136.17	136.17	0.683E-08

Such currency pair, the AUDCAD one, is particularly interesting, as according to the European Central Bank report [48], since the beginning of the financial crisis in 2007, the involvement of non-traditional foreign currencies in international reserves has been tripled. This tendency has been lead by the Canadian dollar (CAD) and Australian dollar (AUD), which represents approximately 25 percent of the non-traditional world reserves. This tendency is consequence of, in one hand, the increase of risk perception in traditional currencies and in the other one, the vigour of the economy from both countries.

**Fig 5:**
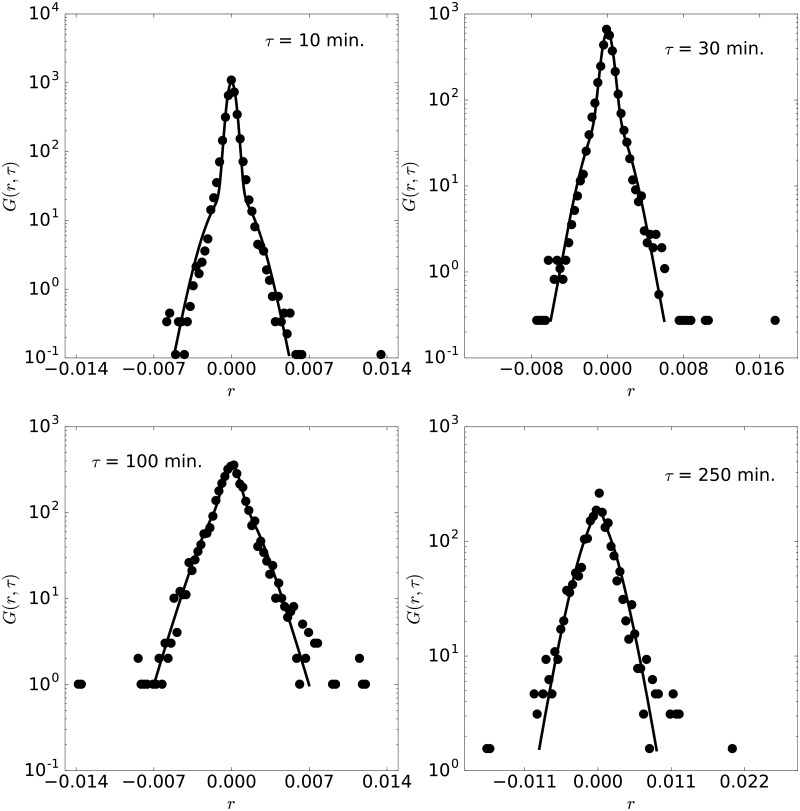
Pdf of EURUSD. Pdf of EURUSD with lag times 10, 30, 100 and 250 minutes, for the year 2015.

Let us focus next on the EURUSD pair, exemplified in Fig 5 showing the pdfs for the year 2015. The fittings of the model are also very satisfactory to all years, capturing again the tails at large variations of the exchange (either positive or negative). The parameters for these fittings are presented in Table 1, second block. Different from the previous case, the time scales for the EURUSD exchange rates show a clear trend, with *τ*_1_ > *τ*_2_ for the period 2010–2013, while *τ*_1_ = *τ*_2_ for 2014–2016, and concomitantly *d* ≳ l. This indicates that the dynamics of the EURUSD is more hindered in the former period than in the latter, coincident with the debt crisis in the Eurozone. Interestingly, the maximum distance between the experimental and theoretical CDF is found for the year 2014, where it goes up to *Er* = 5.5%—for all other years, the difference is below *Er* = 3%.

**Fig 6:**
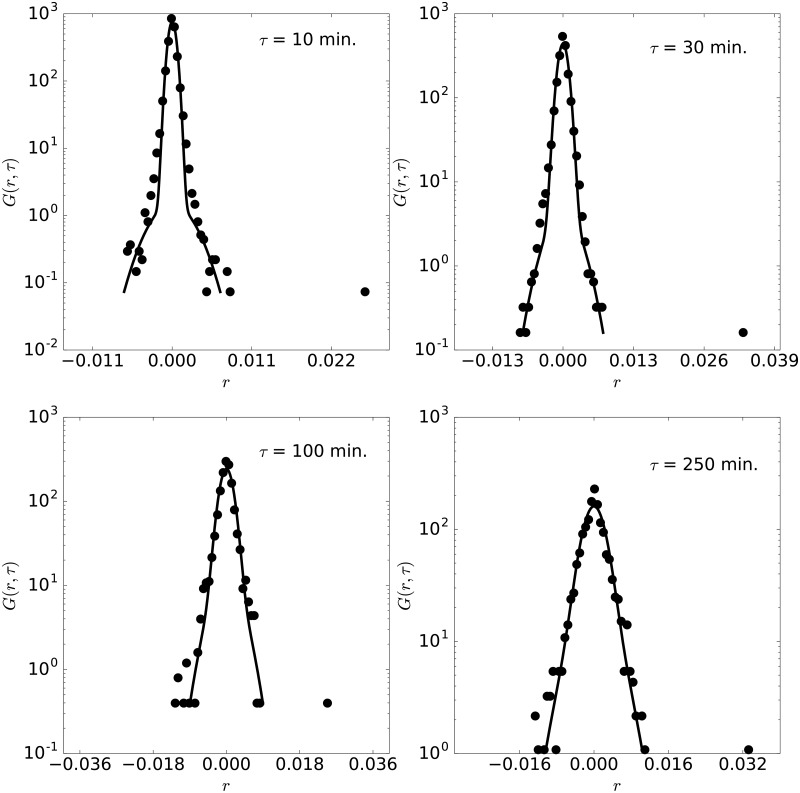
Pdf of GBPJPY. Pdf of GBPJPY with lag times 10, 30, 100 and 250 minutes, for the year 2011.

**Fig 7:**
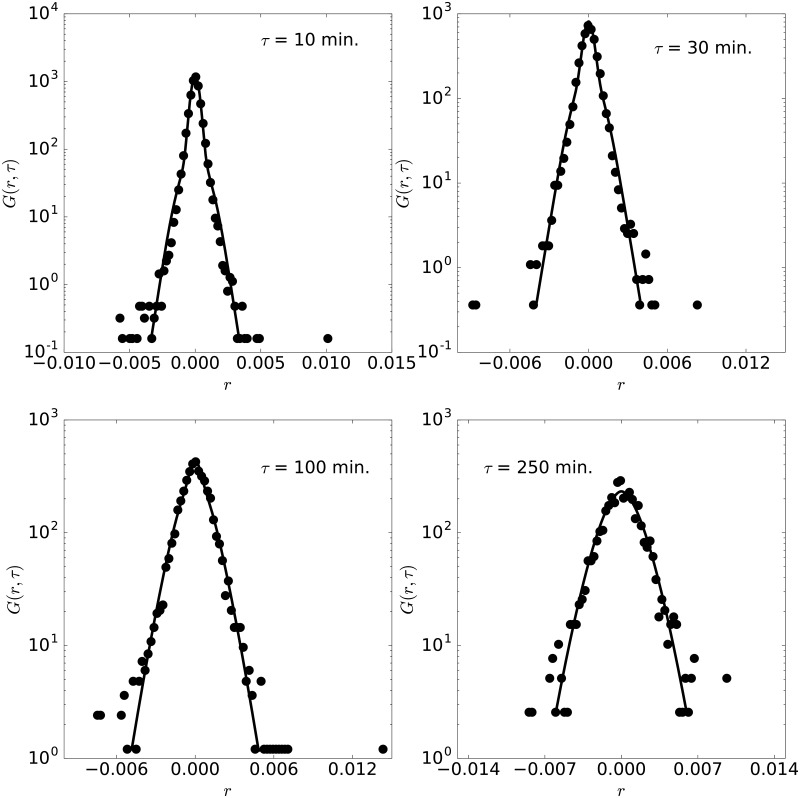
Pdf of NZDSGD. Pdf of NZDSGD with lag times 10, 30, 100 and 250 minutes, for the year 2014.

We now study other currency pairs, as introduced above. Fig 6 shows the GBPJPY rates during 2011. A particularly interesting feature is the large time scales for this year 2011 (Fig 6), *τ*_1_ = *τ*_2_ ≈ 1000 minutes, as the pdf is narrower in this year, although other parameters do not show any specific behavior. The maximum difference is again below *Er* = 4% for all years and lag times. The pdfs belonging to the change pair of the New Zeland and Singapur dollars (NZDSGD) are studied in Fig 7 for 2014. The fittings are also quite satisfactory (the maximum distance below *Er* = 4% for all years and lag times) and the parameters, indicated in Table 1, are again consistent with *τ*_1_ ≈ *τ*_2_. This pair is also considered not conventional and it is formed by the currencies of two countries that belong to the P4 free trade agreement signed in 2005 and that is avoiding 90% of duties gradually.

**Fig 8:**
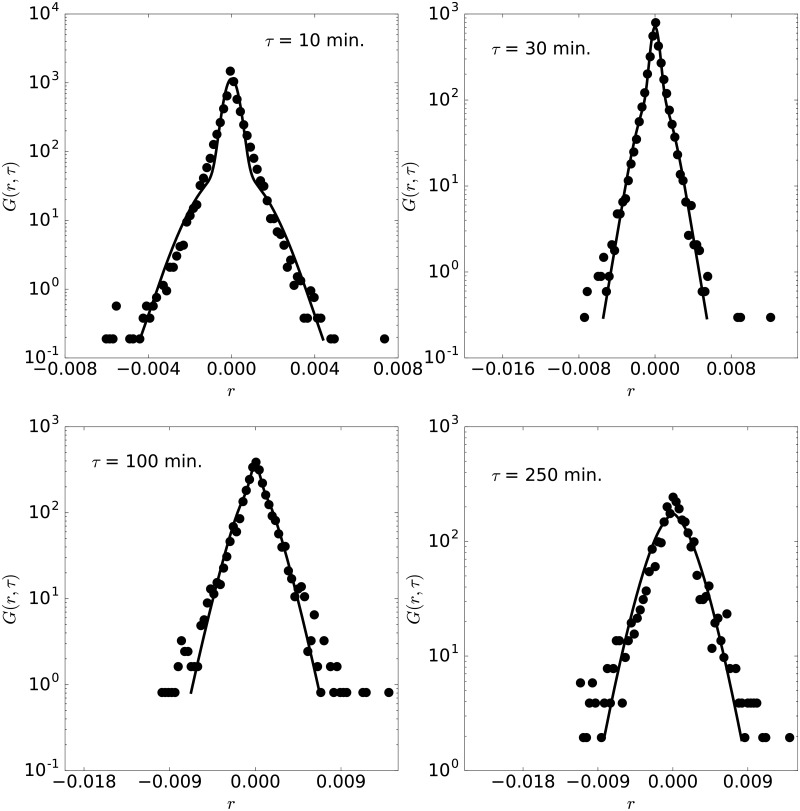
Pdf of USDMXN. Pdf of USDMXN with lags 10, 30, 100 and 250 minutes for the year 2015.

The US dollar—Mexican peso (USDMXN) currency pair is studied as indicated in Fig 8 for the year 2015. The model describes the experimental data with excellent agreement (the maximum distance between experimental and theoretical CDF is below *Er* = 5%), as shown previously for other pairs, with parameters detailed in Table 2, first block. Again, *τ*_1_ ≈ *τ*_2_, due to the intense commercial relation between Mexico and the US.

**Fig 9:**
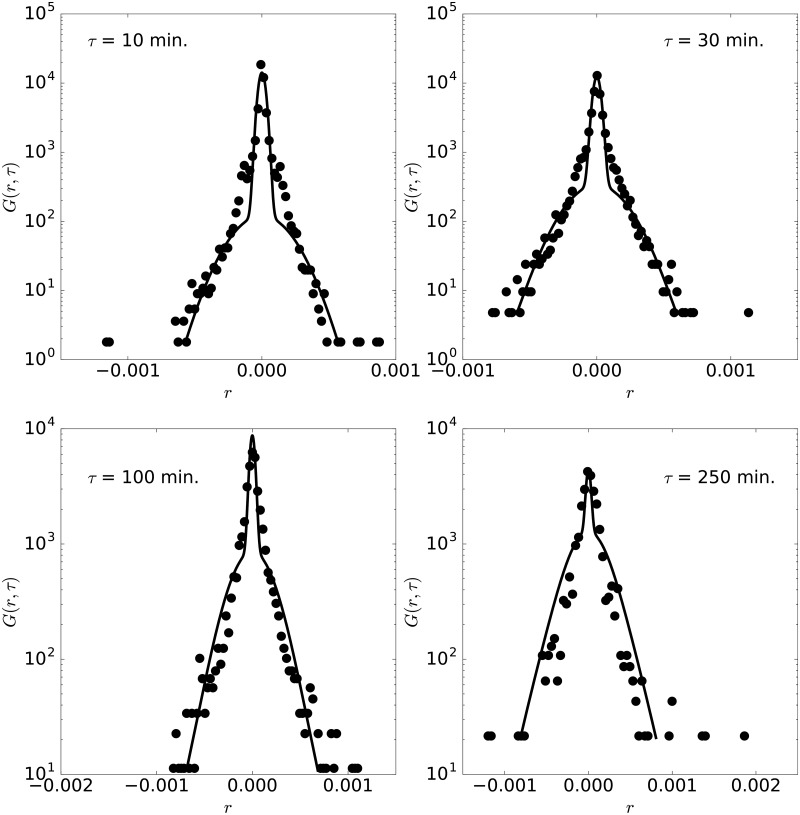
Pdf of USDHKD. Pdf of USDHKD with lags 10, 30, 100 and 250 minutes for the year 2016.

Finally, we study the exchange rate between the US dollar and the Hong Kong dollar, depicted in Fig 9 for the year 2016. This particular year is chosen as an example of an *imperfect* overall fitting, according to our criterion: *Er* = 8.3%, 5.7%, 5% and 10.6% for lag times *τ* = 10, 30, 100 and 250 min., respectively. Still, the fitting is quite satisfactory, and the main features of the distribution are captured. The parameters, given as well in Table 2, yield again *τ*_1_ ≈ *τ*_2_. Interestingly, the pdfs are narrower in this case than in the previous ones. This is captured in our model by the smallness of parameters *l* and *d*, but in all cases *d* is much larger than *l*, indicating that the exchange pair is bracketed in a narrow range, and in a time scale *τ*_1_ ≈ *τ*_2_ it jumps out to a different value. Other currency pairs have been studied, with the corresponding parameters presented in Table 2.

Tables 1 and 2 compile the fitting parameters for all studied currency pairs. Whereas the values of *l* and *d* are non-dimensional parameters, because the log-return is used, the time scales are all measured in the same units, allowing a straightforward comparison between different pairs. Note that both *τ*_1_ and *τ*_2_ are typically in the range of one to three hours, for all pairs. Given the very different currencies studied, this indicates a common origin for the dynamics of the foreign exchange market, irrespective of the particular pair studied. One can think of market makers and short time traders producing the caging process, since they go in and out in their positions, while larger time investors provide transactions on only one side (up or down) of the market. In this context, our analysis indicates that long time investors enter in the market with a time scale of a few hours.

Looking at particular currency pairs, some of them are more stable than other ones. It is interesting to remark the results obtained for the AUDCAD exchange rate, which clearly is the most stable among the years. As mentioned above, this is probably due to both currencies being considered commodity currencies. Other pairs, such as the NZDSGD or the USDHKD, present particular years with different behavior.

**Fig 10:**
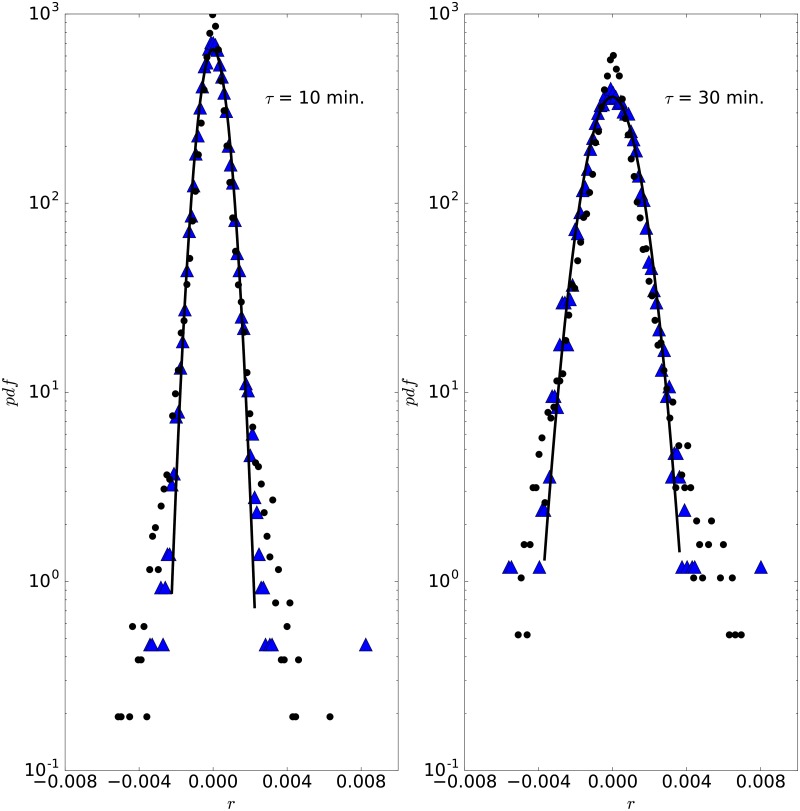
Comparison of the experimental pdf vs iid one. Comparison of the experimental pdf (circle) vs iid one (triangles) for EURUSD in the year 2010 for a lag time of 10 minutes (left panel) and 30 minutes (right panel). The solid line is a Gaussian fit to the iid process.

As the proposed model successfully resolves the experimental pdfs from currency pairs, we study next the experimental data to notice that there is some autocorrelation in the signal, i.e. *r*(*τ*) is not the same as *τ* × *r*(1), see Fig 10. This implies that independently identically distributed pdfs with heavy tails cannot be used to model the log-return distribution of a currency pair. In Fig 10, we can see that the empirical distribution of log-returns with lag times of 10 and 30 minutes is not the same as the distribution of an iid process, featured by log-returns with a lag time of one minute. This is in agreement with Hsieh [49], who concluded that observations for the exchange rate of the US dollar were not independently drawn from a heavy tail distribution that remains fixed over time, but from distributions whose parameters change over time. In particular, in this case, the mean and variance change over time and an ARCH model is able to capture most of the nonlinear stochastic dependencies of the data. Following Hsieh’s finding, other works [50–52] obtained similar results. GARCH formulations by [53–55] went in the same line. With our model, however, we can account for some kind of autocorrelation without the use of additional models.

It can also be noticed in Fig 10 that the empirical distributions are more peaked than the iid process, and that these have heavier tails. This is in agreement with our model, since the Ornstein-Uhlenbeck process, which cages the price, produces a more peaked distribution, while the jump component explains the larger tails. In terms of the market, we can think of market makers and short time traders producing the caged process, since they keep in and out trading positions, while larger time investors provide transactions on only one side (up or down) of the market, accounting for the jump component.

Indeed, as we pointed out in previous sections, foreign exchange markets present some characteristics that make them different from other financial markets, of which the more important ones are that major trading volume is given by market makers, as well as decentralization. Market makers play a fundamental role in prices formation, and considering that these market operators have the obligation of trading at published prices, over which a margin has been fixed, it seems logical to think that they necessarily contribute to engage market price. On the other hand, as [37] showed, it is proved that short term operators and long term ones trade over the base of different expectations. In foreign exchange markets, long term operators, global banks as well as multinational companies, basically make coverture operations for their commercial transactions. Short term traders, on the other hand, play a similar role to market makers since they use stop loss and profit mechanisms based on chartist analysis. Summarizing, as well as [33–35] showed, we think that depending whence the large market trade is coming, from short term or long term traders, the price formation is engaged or not.

## Conclusions

We have proposed a model, derived initially to describe the dynamics of undercooled physical systems, that is able to describe currency pairs with a single functional form, and a single set of parameters for all time lags. More importantly, the parameters can can be physically interpreted, making the model more useful. In particular, the ratio of the two time scales involved in the model, *τ*_1_ and *τ*_2_, indicates if the dynamics of the model corresponds to a high temperature fluid (fast long-time dynamics), or an undercooled system (slow long-time dynamics). We have shown that the model correctly fits many different currency pairs with *τ*_1_ ≈ *τ*_2_, for most cases; the time scales for jumps are in the range of one to four hours, pointing to a common origin in all cases. In agreement with Hsieh [49, 53, 54], Milhoj [52], Diebold [50], Diebold and Nerlove [51], McCurdy and Morgan [56] and Kugler and Lenz [55], our model does not assume the iid restricted condition.

The arrested dynamics found by the model, as well as jumps, could be explained by the previous mentioned heterogeneity of expectations pointed out by classic foreign exchange markets literature (see [32, 36–38, 57–61]). It is suggested that such heterogeneity of expectations is the consequence of the different analysis techniques used by market participants. Traders use information in a different way than portfolio managers and fundamentalists and, in foreign exchange market, one cannot neglect currency coverture operations carried out by international companies. The model presented here does not break the market efficiency hypothesis, but clearly shows how market dynamics transits from arrested, in short term, to diffusive in long term, and we propose, as Engle et al. [62] pointed out, that such behavior is attributed to flow of market information and how market agents process it.

It is interesting to see how not conventional currencies such as AUDCAD and NZDSGD present fits with very stable parameters among the years. In both cases we consider that this is because trade of these currencies is more associated to investments than to speculation.
